# Spatial Transcriptomics in a Case of Follicular Thyroid Carcinoma Reveals Clone-Specific Dysregulation of Genes Regulating Extracellular Matrix in the Invading Front

**DOI:** 10.1007/s12022-024-09798-0

**Published:** 2024-01-27

**Authors:** Vincenzo Condello, Johan O. Paulsson, Jan Zedenius, Anders Näsman, C. Christofer Juhlin

**Affiliations:** 1 Department of Oncology-Pathology, Karolinska Institutet, Stockholm, Sweden; 2 Department of Trauma and Emergency Surgery, Karolinska University Hospital, Stockholm, Sweden; 3 Department of Molecular Medicine and Surgery, Karolinska Institutet, Stockholm, Sweden; 4 Department of Breast, Endocrine Tumors, and Sarcoma, Karolinska University Hospital, Stockholm, Sweden; 5 Department of Pathology and Cancer Diagnostics, Karolinska University Hospital, Stockholm, Sweden

**Keywords:** FTC, Invasion, Spatial transcriptomics, Phylogenetics, Extracellular matrix

## Abstract

Follicular thyroid carcinoma (FTC) is recognized by its ability to invade the tumor capsule and blood vessels, although the exact molecular signals orchestrating this phenotype remain elusive. In this study, the spatial transcriptional landscape of an FTC is detailed with comparisons between the invasive front and histologically indolent central core tumor areas. The Visium spatial gene expression platform allowed us to interrogate and visualize the whole transcriptome in 2D across formalin-fixated paraffin-embedded (FFPE) tissue sections. Four different 6 × 6 mm areas of an FTC were scrutinized, including regions with capsular and vascular invasion, capsule-near area without invasion, and a central core area of the tumor. Following successful capturing and sequencing, several expressional clusters were identified with regional variation. Most notably, invasive tumor cell clusters were significantly over-expressing genes associated with pathways interacting with the extracellular matrix (ECM) remodeling and epithelial-to-mesenchymal transition (EMT). Subsets of these genes (*POSTN* and *DPYSL3*) were additionally validated using immunohistochemistry in an independent cohort of follicular thyroid tumors showing a clear gradient pattern from the core to the periphery of the tumor. Moreover, the reconstruction of the evolutionary tree identified the invasive clones as late events in follicular thyroid tumorigenesis. To our knowledge, this is one of the first 2D global transcriptional mappings of FTC using this platform to date. Invasive FTC clones develop in a stepwise fashion and display significant dysregulation of genes associated with the ECM and EMT — thus highlighting important molecular crosstalk for further investigations.

**Supplementary Information:**

The online version contains supplementary material available at 10.1007/s12022-024-09798-0.

## Introduction

Accounting for approximately 10–15% of thyroid cancer diagnoses overall, follicular thyroid carcinoma (FTC) ranks as the second most common form of malignancy arising in the thyroid gland [1]. Although most FTCs may exhibit an indolent clinical course, some show hematogenous spread to distant organs, like lungs and bones [2–4].

FTC represents the malignant *alter ego* of the benign follicular thyroid adenoma (FTA) [5]. The diagnostic process of the FTCs primarily revolves around histologically demonstrating invasive properties in excised tissue. Therefore, the presence of capsular or vascular invasion serves as definitive criteria for diagnosing FTC, which distinguishes the malignant from the benign form. [6]. These microscopic attributes cannot be determined through an initial fine needle aspiration cytological (FNAC) examination. These tumors, indeed, exhibit not only similar morphologic characteristics to FTA but also common genetic and immunohistochemical profiles. Only a few recurrent differences appear in the somatic landscape of these lesions — clearly not yet sufficient to distinguish these two entities by either immunohistochemistry or molecular genetics pre-operatively [7, 8]. Given the similar features, distinguishing these two entities before surgery still represents one of the most difficult diagnostic challenges in thyroid pathology. In light of this limitation, most patients with indeterminate cytological findings after FNAC are usually offered diagnostic lobectomy to obtain the correct diagnosis [9, 10].

Previous research in FTCs has identified regional genetic variances, suggesting subclonal expansions from a mother clone. For example, early studies utilizing comparative genomic hybridization techniques in a single FTC revealed genomic alterations at the chromosomal level that were exclusive to specific geographical regions, also identifiable through microscopic investigations of different histological growth patterns [11]. Expressional differences in various transcription factors have also been reported near the invasive fronts of FTCs, potentially indicating subtle events of interest related to the tumor’s acquisition of malignant potential [12].

Moreover, on the pan-genomic level, subclones in FTCs have been found to progress into more biologically aggressive entities, such as poorly differentiated thyroid carcinoma (PDTC) and anaplastic thyroid carcinoma (ATC), suggesting that the genetic heterogeneity may be of clinical importance to highlight the potential for dedifferentiation [13, 14]. As an example, *TERT* promoter mutations are genetic events associated with adverse outcomes in thyroid cancer and are considered subclonal events in low-risk follicular thyroid neoplasms and well-differentiated thyroid carcinoma, but clonal in PDTCs and ATCs — again arguing for the development of subclonal expansions in FTCs that may signal aggressive behavior [15–17].

In this study, we aimed to analyze an FTC using spatial transcriptomics (ST) to provide a more comprehensive mapping of any geographical differences in gene expression. Gene expression patterns coupled to invasive behavior were specifically sought and validated in an extended set of FTCs, and tumoral phylogenetics was studied to visualize the clonal expansions.

## Materials and Methods

### Sample Collection

With the approval of the Swedish Ethical Review Authority (EPN 2015_959-31), tumor samples were obtained from the Karolinska University Hospital, Stockholm, Sweden. All cases were reviewed to confirm the histological diagnosis by an endocrine pathologist (C.C.J.). For the index case used for ST analysis, we selected an encapsulated angioinvasive FTC recently diagnosed in our department. For this specific case, vascular invasion was extensive, with >4 areas with clear-cut venous invasion. Capsular invasion was limited, with only two foci. There were no blocks available in which vascular and capsular invasion co-existed, so the most representative areas with clear-cut capsular and vascular invasion, respectively, as well as a capsule-near area without invasion, and a central area of the tumor without an associated capsule, were selected for ST analysis. The vascular invasive features were verified using endothelial markers as well as platelet-related marker CD61 to rule-out artefactual intravascular tumor deposits [18]. Demographic information including age, sex, and nodule size was collected at the time of diagnosis.

Moreover, an independent cohort of 30 follicular thyroid tumors (20 FTCs and 10 FTAs) diagnosed in 2022–2023 at our hospital was selected for validation analysis using immunohistochemistry. FTCs were subtyped into 14 minimally invasive FTCs (MIFTC), 5 encapsulated angioinvasive FTCs (EAIFTC), and 1 widely invasive FTC (WIFTC).

### Mutational Analysis

Following DNA extraction and purification (QIAamp DNA Mini Kit, Qiagen, Hilden, Germany), the case selected for ST was interrogated for known hotspots of thyroid-related genes using an NGS panel adopted routinely in clinical practice (Oncomine Childhood Cancer Research Assay, Thermo-Fisher Scientific, Waltham, MA, USA). *PAX8::PPRG* fusion analysis was carried out by PCR analysis.

### Spatial Transcriptomics Profiling

Hematoxylin and eosin (H&E)–stained images from tumor samples were examined by one of the authors (C.C.J.). Regions featuring capsular and vascular invasion, an area near the capsule without invasion, and the central core of the tumor were selected. The four representative FFPE tissue blocks were cut and extracted for RNA. The RNA content from all tissue blocks was analyzed to ensure it met the RNA quality threshold (DV200 > 50%) for ST analysis. Following this, the four H&E slides were annotated in terms of regions of interest, ensuring inclusion in the 6 × 6-mm square area employed for ST analysis. One unstained 4-μm-thick section from each block was then deparaffinized and de-crosslinked, and tissue imaging within a 6 × 6-mm square of each section was performed according to the manufacturer’s instructions. Libraries were constructed using Visium CytAssist Spatial Gene Expression for FFPE assay (10× Genomics, Pleasanton, CA, USA) and sequenced on the Illumina NovaSeq 6000 platform to achieve a depth of at least 75,000 mean read pairs and 2000 median genes per spot. The SpaceRanger count pipeline (v2.0.1) was run using the GRCh38 transcriptome data from 10× Genomics to process FASTQ files. Both Loupe Browser’s Manual CytAssist Image Alignment and Manual Fiducial Alignment workflows were used.

### Differential Expression Analysis and Gene Ontology Analysis

Two areas of the index case, one with capsular invasion and the other with vascular invasion, were selected for spatial differential gene expression analysis. Clustering and differential expression analyses were performed using the Loupe Browser (v7.0.1, 10× Genomics). Each spot barcode was clustered using an automated clustering algorithm where clustered spots have similar expression profiles compared to all other spots in the tissue slide (“globally distinguishing”). Differentially expressed genes (DEG) in the different clusters were presented as log2 fold change. *P*-values were based on a negative binominal test and adjusted for multiple tests with the Benjamini-Hochberg procedure. The top 20 most DEG and the top 20 upregulated genes in selected clusters in the invasive front were analyzed for gene ontology (GO) using Enrichr (v 2.1, Jawaid, 2019). We then annotated the capsular/vascular invasion areas and performed differential expression analysis by annotating and comparing with the spots in the tumor tissue outside the invasive front. The top 20 most DEG were analyzed for GO.

### Pseudotime Trajectory Analysis

The pseudotime trajectory analysis was performed using the Monocle3 package with tumor cells defined as root node within Partek^®^ Flow^®^ software, v10.0. Gene set enrichment analysis (GSEA) was performed using the KEGG database within the Partek^®^ Flow^®^ software, v10.0.

### Immunohistochemical Staining

For the index case, all immunohistochemical stainings procured as part of clinical routine analysis were assayed using an automated Ventana approach, and detailed information of the specific antibodies used to diagnose this case is available upon request.

To verify our initial observations from the ST analysis, an immunohistochemical analysis of a validation set was performed to explore and validate specific protein expression in the invading front of FTC compared to FA. Thus, FFPE tissues from a cohort of 20 FTCs (including the index case) and 10 FTAs cases were cut into 4-μm-thick sections followed by deparaffinization in xylene and rehydration in ethanol. All sections were subjected to antigen retrieval in tris-EDTA buffer, pH 8.0 (Sigma, E-1161) at 95 °C for 15 min. Slides were incubated at room temperature with hydrogen peroxide, followed by a 15-min blocking step using Background Sniper (BS966; Biocare Medical). Primary antibodies anti-POSTN (1:400; ab14041, Abcam) and anti-DPYSL3 (1:300; PA552760, Life Technologies) were diluted in Renoir Red diluent (PD904; Biocare Medical) and incubated at 4 °C overnight. According to the manufacturing protocol, the MACH-1 Universal HRP-Polymer Detection kit (M1U539; Biocare Medical) was used for the detection step. Counterstaining with hematoxylin as well as dehydration in ethanol and xylene was performed.

The level of immunoreactivity was evaluated and scored with arbitrarily assigned cutoffs as either negative: (score “0”; <10% of cells showing expression), moderately positive (“score 1”; 10–74% of cells showing expression), and strongly positive: (“score 2”; >75% of cells showing expression). Also, immunoreactivity was separately analyzed in central parts of the tumor, in capsule-near areas, and in invasive areas (the latter only for FTCs). Staining intensity in each part of the tumor was compared to evaluate potentially significant differences.

### Statistical Analysis

Differential expression analysis was performed using Loupe software (v.7.0.1, 10X Genomics, Pleasanton, CA, USA). *P*-values were adjusted with the Benjamini-Hochberg method and false discovery rate (FDR) <0.05 was considered significant. Fisher’s exact test and Kruskal-Wallis multiple comparison tests were used to compare the different staining areas of the tumors. *P*-values <0.05 were considered significant. Statistical computations were performed using GraphPad Prism version 10.1.0 (GraphPad Software, San Diego, CA, USA).

## Results

### Clinical and Molecular Features of the Index Case

**Fig. 1:**
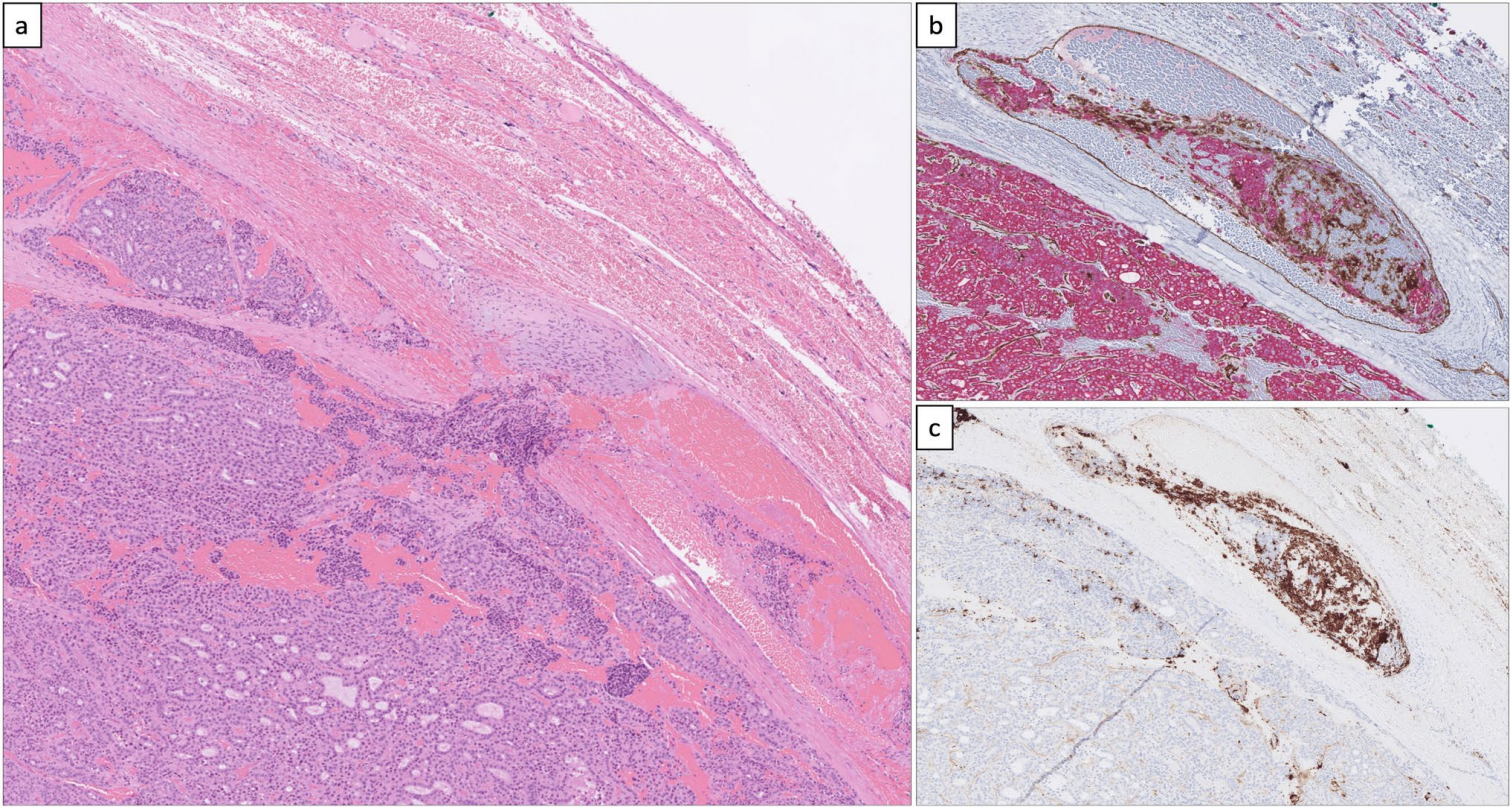
Histological attributes of the index case. **a** Hematoxylin-eosin-stained section depicting extensive vascular invasion. **b** Double stain (CD31 in brown, pan-cytokeratin in red) visualizing intravascular tumor deposits. **c** CD61 immunohistochemistry confirms an *in vivo* phenomenon with thrombus

The index case was a 45-mm encapsulated angioinvasive FTC derived from a 54-year-old female patient (Fig. 1a). The diagnosis was made using the 2022 WHO classification criteria, and differentiated high-grade thyroid carcinoma could be excluded as the mitotic index was 1/2 mm^2^, and no tumor necrosis was evident despite extensive sampling. Venous invasion was observed multifocally, and capsular invasion was also noted. There was no extrathyroidal extension, and surgical margins were negative. Tumor cells were positive for TTF1, PAX8, and partially for thyroglobulin (50% of tumor cells). The Ki-67 index was 4.2%. Tumor cells were negative for BRAF VE1, and the P53 staining was heterogenous and therefore not indicative of an underlying *TP53* gene mutation. Areas with vascular invasion were confirmed using anti-CD31 and anti-CD61 antibodies to highlight endothelial cells and thrombus material, respectively (Fig. 1b, c). The background thyroid tissue showed evidence of thyroid follicular nodular disease, but no other tumors were found. There was no sign of thyroiditis.

The clinical NGS analysis showed a *TERT* C250T promoter mutation. No mutations were detected in the known hotspots of various thyroid-related genes, such as the *RAS* gene family, *BRAF*, *RET*, *MET*, *DICER1*, *PTEN*, *AKT*, *PIK3CA*, *APC*, *MTOR*, and *CDKN2A/B* genes. No *BRAF*, *RET*, *NTRK1/3*, or *ALK* fusions were noted either. PAX8::PPARG fusions were investigated separately using PCR, with negative results.

### Spatial Transcriptomics Profiling to Identify Intra-tumor Heterogeneity of Invasive Properties in FTC

**Fig. 2:**
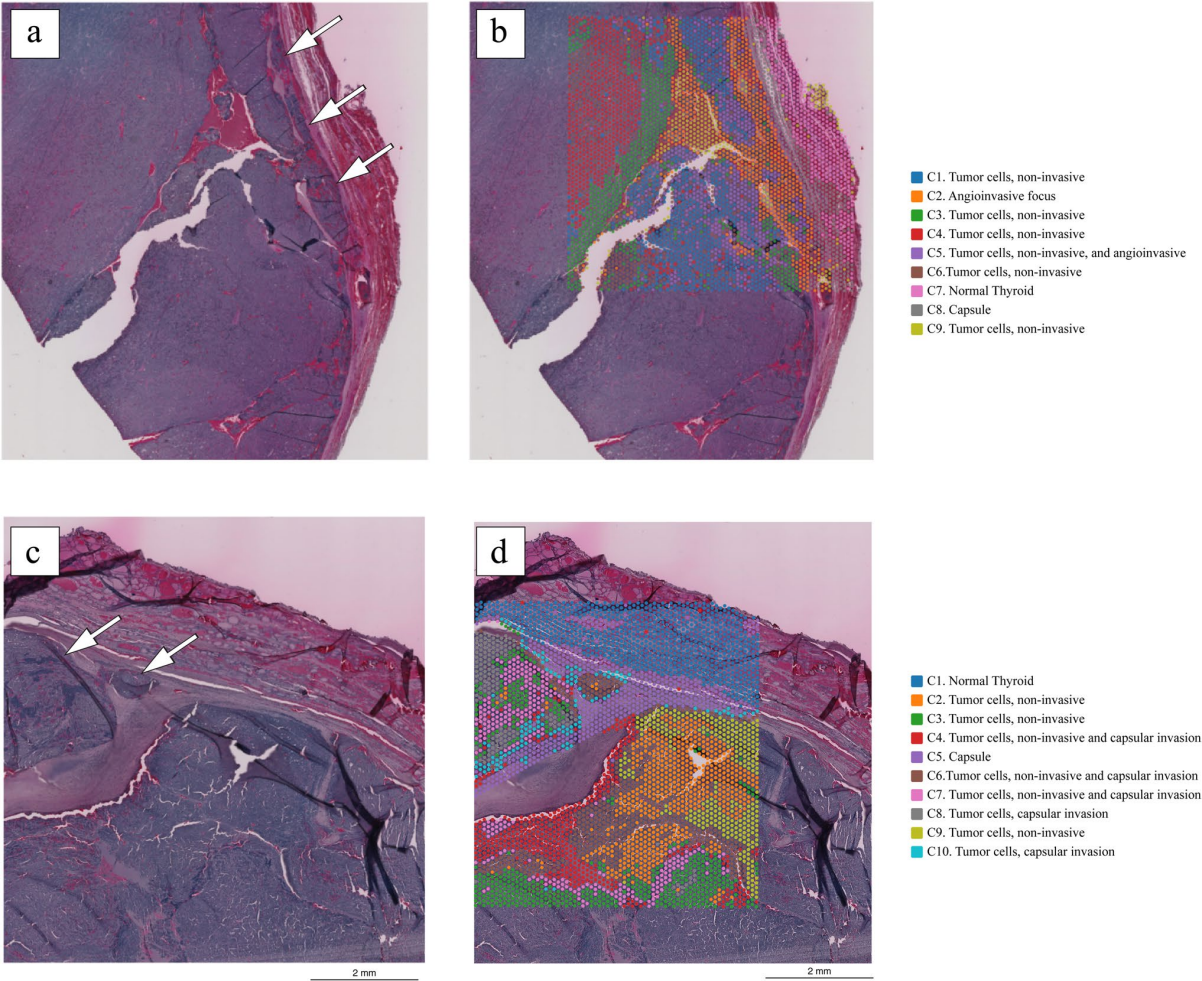
Outline of the spatial transcriptomics analysis. Tiled versions of the two tissue images showing invasion that were used for spatial transcriptomics analysis are shown in **a**–**d**. **a** Hematoxylin-eosin-stained section of the vascular invasion area; arrows indicate the invading foci. **b** Unsupervised clustering analysis showing nine different clusters in slide 1 with vascular invasion. Each color represents a distinct cluster. **c** Hematoxylin-eosin-stained section of the capsular invasion area; arrows indicate the invading foci. **d** Unsupervised clustering analysis showing 10 different clusters in slide 2 with capsular invasion. Each color represents a distinct cluster (C1–10). Each spot within the 6 × 6-mm square represents an area with gene expression information, allowing for gene expression–based clustering information

To comprehensively resolve the molecular heterogeneity during the invasion process in FTCs, we performed ST on four FFPE sections (of which one showed capsular invasion, one vascular invasion, and two without invasion foci) using the 10× Genomics Visium platform (Fig. 2a, c). Transcriptomes from a total of 18,074 spots were sequenced. Detailed sequencing parameters are listed in Supplemental Table 1.

**Table 1:** Summary of the immunohistochemical staining score of the validation cohort

		**POSTN**			**DPYSL3**		
Sample ID	Diagnosis	Invasive front	Tumor periphery	Central tumor	Invasive front	Tumor periphery	Central tumor
1	EAIFTC	0	1	0	1	1	1
2	MIFTC	0	0	1	1	1	1
3	MIFTC	1	1	0	1	1	0
4	MIFTC	1	1	0	1	1	0
5	MIFTC	1	1	1	1	1	1
6	EAIFTC	1	1	1	2	2	1
7	MIFTC	1	1	1	1	1	1
8	MIFTC	1	1	1	1	1	1
9	MIFTC	0	1	0	2	2	0
10	EAIFTC	1	1	1	2	2	1
11	MIFTC	1	1	1	1	1	0
12	MIFTC	2	2	1	1	1	1
13	MIFTC	1	1	0	1	1	0
14	EAIFTC	1	1	0	1	1	0
15	EAIFTC	0	1	0	1	1	1
16	MIFTC	1	1	1	1	1	0
17	MIFTC	1	1	1	1	1	1
18	MIFTC	1	1	1	2	2	1
19	MIFTC	1	1	0	1	1	0
20	WIFTC	1	1	0	1	1	1
21	FTA	na	1	1	na	1	1
22	FTA	na	1	1	na	1	1
23	FTA	na	1	1	na	1	0
24	FTA	na	0	0	na	0	0
25	FTA	na	1	1	na	1	1
26	FTA	na	1	0	na	1	0
27	FTA	na	1	1	na	1	1
28	FTA	na	1	1	na	1	0
29	FTA	na	1	1	na	1	1
30	FTA	na	1	1	na	1	1

The level of immunoreactivity was evaluated and scored as either negative: score “0”, (<10% of cells showing expression), moderately positive: score “1” (10–74% of cells showing expression), and strongly positive: score “2” (>75% of cells showing expression)

*FTA* follicular thyroid adenoma, *MIFTC* minimally invasive follicular thyroid carcinoma, *EAIFTC* encapsulated angioinvasive follicular thyroid carcinoma, *WIFTC* widely invasive follicular thyroid carcinoma; *na* not applicable

### Unsupervised Clustering Reveals Distinct Clusters in Invading Fronts

After identifying and annotating the morphological tumor spots, we performed an unsupervised clustering to unravel the spatial heterogeneity across the tumor. In the case with vascular invasion (slide 1), 9 different clusters were identified across the tumor, stroma, and adjacent normal tissue (Fig. 2b), while in the case with capsular invasion (slide 2), 10 different clusters were identified (Fig. 2d).

In slide 1, clusters 2 (orange) and 5 (purple) were enriched in the vascular invasion area (Fig. 2b). The top 20 DEG in cluster 2 showed significant enrichment in the GO biological process “Negative Regulation of Smooth Muscle Cell Migration” (GO:0014912) and GO cellular component “Collagen-Containing Extracellular Matrix” (GO:0062023), whereas cluster 5 showed enrichment in GO biological process “Collagen Fibril Organization” (GO:0030199) and GO cellular component “Collagen-Containing Extracellular Matrix (GO:0062023)” (Supplemental Table 2).

In slide 2, several clusters occurred both in the invasive front and central parts of the tumor; however, clusters 8 (gray) and 10 (light blue) were almost exclusively enriched in the capsular invasion area. Cluster 6 (brown) occurred in the central part of the tumor but was also intensely enriched in a separate capsular invasion area surrounded by a few spots from cluster 10 (Fig. 2d). The top 20 DEG in cluster 6 were significantly enriched in GO biological process “Extracellular Matrix Assembly” (GO:0085029). Furthermore, both clusters 8 and 10 showed enrichment in GO biological process “Extracellular Matrix Organization” (GO:0030198) (Supplemental Table 2). DEG in capsular/vascular invasion areas *versus* the central part of the tumor also showed enrichment in GO associated with extracellular matrix components and processes.

Among the most upregulated genes in clusters involved in the invasive areas and with biological relevance to tumorigenesis such as EMT, ECM, or tumor invasion, we selected two genes for further analysis and validation. In particular, *DPYSL3* was the most upregulated gene in cluster 6 of slide 2 (Supplemental Fig. 1a–b), whereas *POSTN* was the most significantly up-regulated gene in cluster 10 of slide 2 and ranked in the top 5 most upregulated gene in cluster 2, slide 1 (Supplemental Fig. 1c–d).

The two areas without invasive features (slides 3 and 4) were also analyzed for differences in gene ontology, in which clones positioned near the tumoral capsule were enriched for dysregulation of genes associated to “Elastic Fiber Assembly” (GO:0048251), “Supramolecular Fiber Organization” (GO:0097435), and “Extracellular Matrix Assembly” (GO:0085029) compared to more centrally located clones (data not shown).

Furthermore, the identification of a *TERT* promoter mutation prompted us to investigate the expression levels of the *TERT* gene throughout the tumor. The analysis showed the existence of a few spots disseminated across the tumor tissue, mostly reaching both the central core and the periphery/invasive front of the tumor, suggesting an irregular expressional pattern of this gene in the index case (Supplemental Fig. 2).

### Pseudotime Trajectory Analysis Reveals Angioinvasion and Epithelial-Mesenchymal Transition (EMT) as Late-Stage Events

**Fig. 3:**
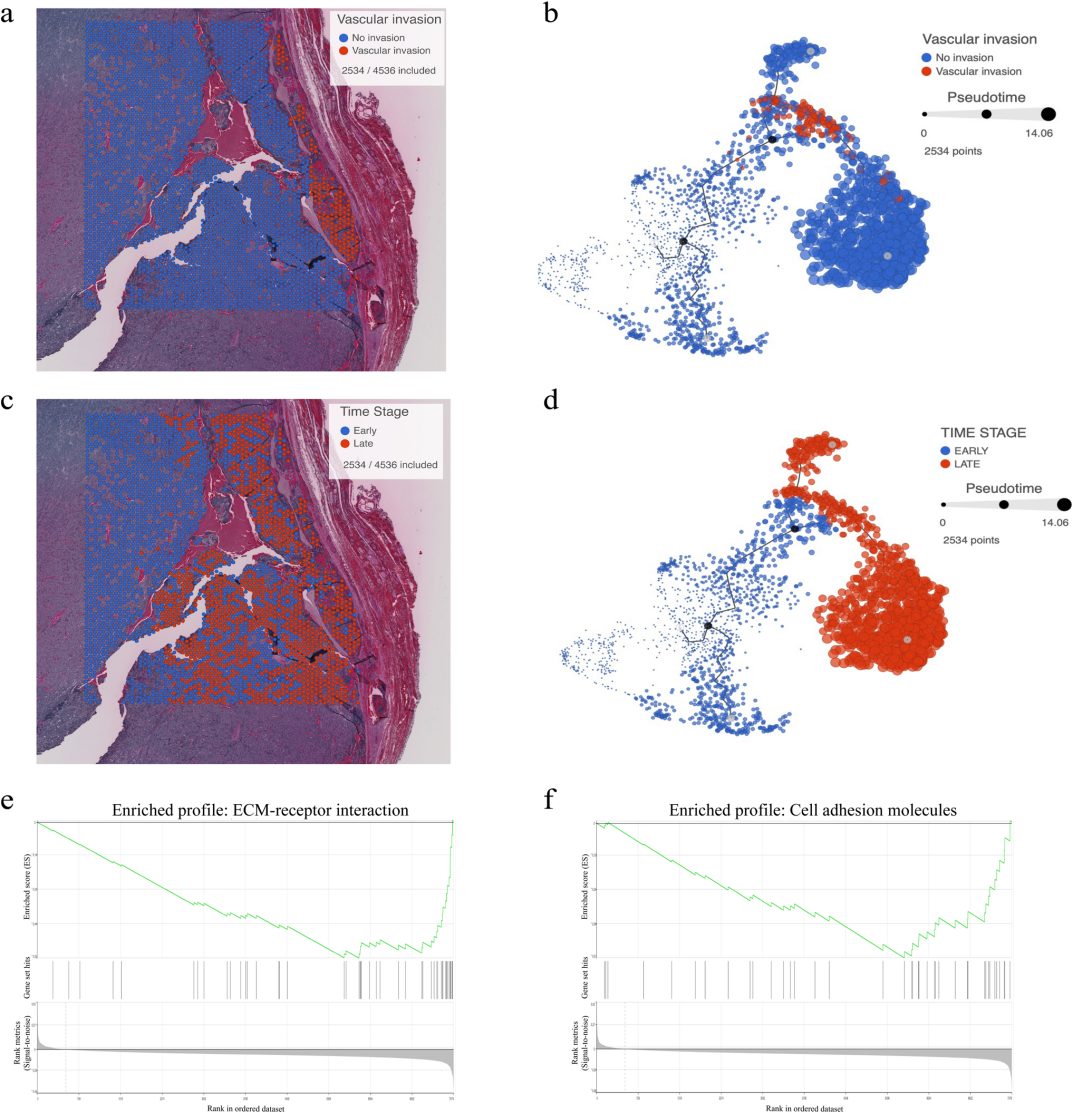
Trajectory analysis and gene set enrichment analysis in the angioinvasive tissue sample. **a** Hematoxylin-eosin-stained section of angioinvasive tissue with filtered tumor cell spots for analysis. **b** Trajectory analysis with tumor cell spots, showing clustering of angioinvasive tumor cell spots occurring with high pseudotime. **c** Hematoxylin-eosin-stained section of angioinvasive tissue representing the time stage of “early” and “late” tumor cells. **d** Trajectory analysis dichotomized into “early” and “late” tumor cells based on median pseudotime. **e** Gene Set Enrichment Analysis (GSEA) plots demonstrating downregulation of ECM-receptor interaction (path:hsa04512) in early versus late (NES −2.03, *p*-value 0.00, FDR 0.00). **f** Gene set enrichment analysis (GSEA) plots showing the downregulation of cell adhesion molecules (path:hsa04514) in early versus late (NES −0.51, *p*-value 0.00, FDR 0.05)

To gain insight into the spatial locations and to unravel spatiotemporal patterns of invasive cells, a trajectory analysis was performed on the tissue case showing angioinvasion (slide 1) after having filtered for tumor cells only by histomorphology and manual annotation of the tumor spots (Fig. 3a). The trajectory analysis revealed that tumor cells with vascular invasion clustered together and seemed to have a high pseudotime (Fig. 3b). Furthermore, after having dichotomized the tumor cells into “early” and “late” tumor cells, based on the median pseudotime (Fig. 3c, d), a gene set enrichment analysis (GSEA) was performed. In total, six gene sets were significantly differentially enriched between early and late tumor cells (Supplemental Table 3). Notably, two out of these six gene sets were associated to EMT (ECM-receptor interaction and cell adhesion molecules) (Fig. 3e, f). Both gene sets were negatively enriched in early *versus* late tumor cells.

### Distinct Expression Gradient of Extracellular Matrix (ECM)–Related Markers from Tumor Center to Periphery

Two of the DEG in the invasive clusters were selected to verify and validate our initial observations shown by ST analysis. Immunohistochemical staining for cytoplasmic proteins DPYSL3 and POSTN was performed in an independent cohort of 20 FTCs and 10 FTAs.

**Fig. 4:**
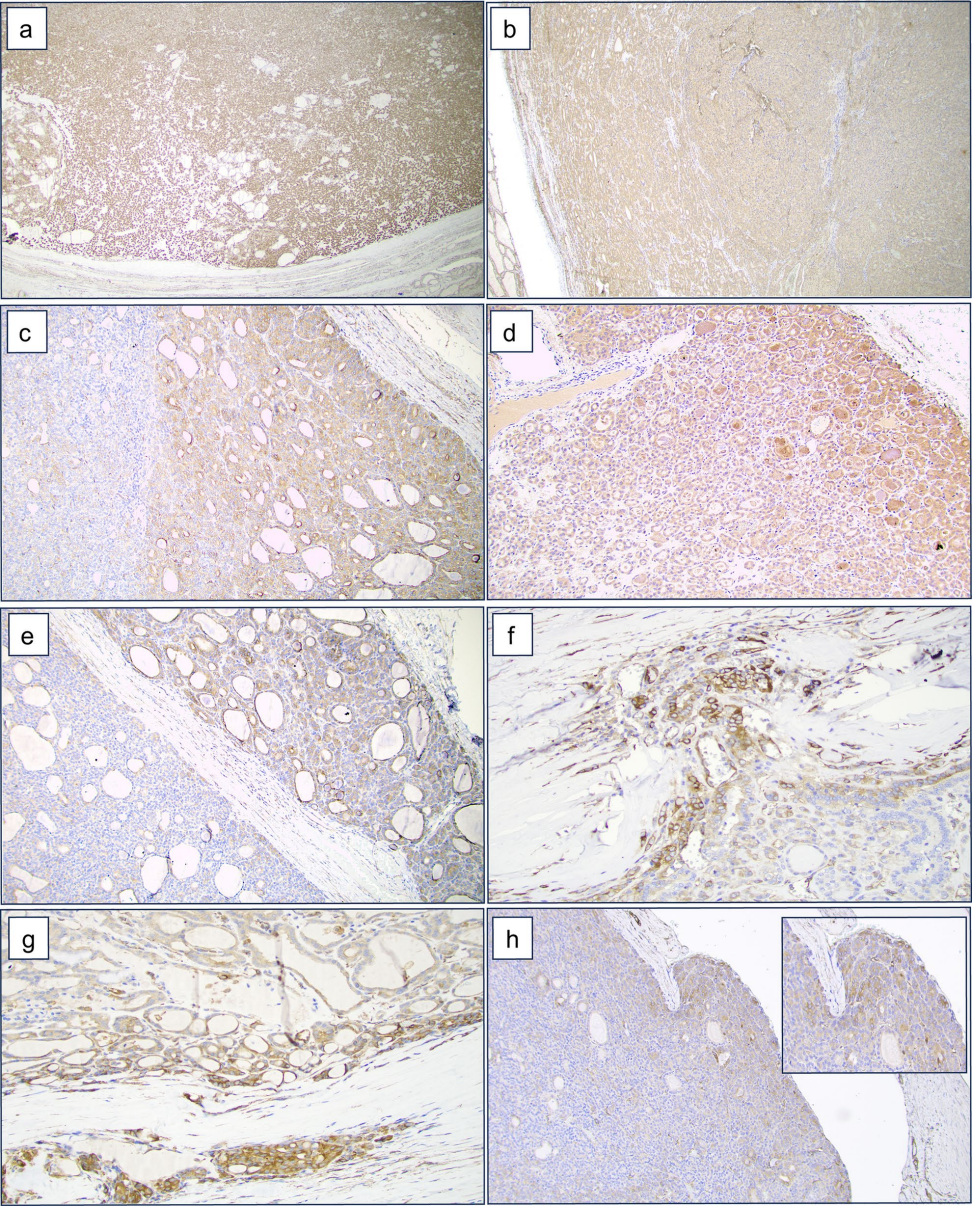
Extracellular matrix (ECM) marker immunohistochemistry. Immunohistochemical staining of DPYSL3 and POSTN in follicular thyroid carcinoma (FTC) compared to follicular thyroid adenoma (FTA). **a**, **b** Staining showing the homogeneous expression of DPYSL3 and POSTN respectively in FTA. **c**, **d** Pronounced expression of DPYSL3 and POSTN, respectively, characterized by increased intensity extending from the central region towards the periphery of the FTC. **e**, **f** Strong expression of DPYSL3 and POSTN respectively in the area with capsular invasion. Statistical significance for this feature was observed only for DPYSL3. **g**, **h** Pronounced expression of DPYSL3 and POSTN in the area of vascular invasion. Statistical significance for this feature was observed only for DPYSL3

The staining revealed an exclusive cytoplasmic localization for both markers. The level of immunoreactivity of both proteins in FTAs was homogeneous throughout the tumor, both in the central core and periphery of the tumor. Among the 10 FTAs stained for POSTN, 8 cases received a score of 1 in the central part of the tumor, while 9 were scored 1 in the periphery. Similar results were obtained with DPYSL3: 6 cases scored 1 in the central part of the tumor, while 9 obtained a score of 1 in the periphery. None of the 10 FTAs stained for both markers received a score of 2 (Table 1). No significant differences were found in comparing the central part *versus* the periphery of the tumor for both proteins (Fig. 4a, b).

Among the 20 FTCs stained for DPYSL3, 12 cases (60%) exhibited central tumor staining (score 1). In contrast, all 20 cases (100%) stained for DPYSL3 displayed a distinct expression pattern, characterized by heightened intensity extending from the central region of the tumor towards the periphery, particularly in proximity to the tumor capsule. The same expression pattern was observed in FTCs stained for POSTN, where 11 cases (55%) showed central tumor staining (score 1), and 19 cases (95%) exhibited moderate to strong positivity, particularly near the tumor capsule (Table 1; Fig. 4c, d). Significant differences were noted when comparing the immunoreactivity levels of the central core part *versus* the periphery of the tumor (*p* = 0.001) for both DPYSL3 and POSTN proteins. Moreover, strong cytoplasmic staining was also evident in areas exhibiting capsular (Fig. 4e, f) and vascular invasion (Fig. 4g, h). All 20 cases (100%) stained for DPYSL3 showed moderate or strong expression intensity around the capsular or vascular invasion focus, showing a significant difference compared to a more central part of the tumor (*p* = 0.002). Sixteen out of 20 FTCs (80%) stained for POSTN exhibited moderate or strong expression intensity around the capsular or vascular invasion focus; however, no significant differences were found in comparing the central core *versus* the invasive front of the tumor.

## Discussion

Gene expression profiling offers significant insights into evolutionary processes within a tumor. In our case, spatial transcriptomics analyses were employed to increase our understanding of intricate biological processes by incorporating spatial context into gene expression data, allowing for visualization of clonal composition in an encapsulated angioinvasive FTC.

This work aimed to identify clonal differences between invasive and non-invasive areas and construct a timeline to visualize whether invading clones develop early or late in thyroid carcinogenesis. Interestingly, our results highlight the presence of distinctive tumor clones in regions infiltrating the tumoral capsule and vascular spaces. These clones exhibit an enrichment in genes targeting the EMT and ECM, a finding corroborated by gene ontology analyses comparing invading clones to non-invading clones. Two of these genes were also further investigated on the protein level, showing a significant association between central and capsule-near areas in an independent set of tumors. Furthermore, a phylogenetic tree constructed for an area with vascular invasion aided in identifying invading cells as relatively late events compared to the more central parts of the tumor. This suggests that clones of FTC develop from the central regions outward.

The role of spatial heterogeneity in FTCs is still poorly understood. Nevertheless, earlier work has identified regional variances in transcription factor expression patterns near the invasive fronts. For example, the proteins YY1 and MAX have been found to be significantly overexpressed at the tumor periphery, most notably in the area of invasion [12]. In our study case, the peripheral and invasive clones exhibited enrichment for ECM interacting genes compared to more centrally located clones. This aligns with the assumption that invasive tumor cells stimulate ECM remodeling or degradation to traverse the capsule or enter the vascular space [19]. Indeed, previous studies have identified implications for ECM degradation as significant contributors to the invasive potential of FTCs, including dysregulation of matrix metalloproteinases (MMPs) and the plasmin activation system [20–22]. Interestingly, cell adhesion pathways were also up-regulated at the invasive fronts. The reason for the up-regulation of cell adhesion-related genes in the tumor periphery is not known, but it is known that cancer cells may require sufficient cell adhesion during the degradation of the ECM to avoid anoikis, a special type of apoptosis triggered in cells detached from the ECM [23].

In our validation cohort, we observed a notable overexpression of POSTN and DPYSL3 proteins in proximity to the capsule compared to the central core areas of FTCs. In FTAs, this trend was less apparent, with a consistent, non-varying, expression of these proteins observed throughout the nodule, both in the central and peripheral regions of the tumor. This observation suggests a potential involvement of the regional expression of these two proteins in the invasion process. Particularly interesting, *POSTN* (also known as *Periostin*) is a gene that encodes a secreted protein that binds to integrins to aid in adhesion and migration of epithelial cells. Recently, several studies have shown interest in this gene, reporting the crucial role of *POSTN* in tumor growth, angiogenesis, invasiveness, and metastasis [24–26]. In papillary thyroid carcinoma (PTC), POSTN has been shown to be upregulated in the invasive front and seems to correlate with aggressive behavior [27, 28]. Similar results were reported in FTCs by Kusafuka et al*.* who demonstrated strong POSTN expression in the sclerotic stroma of the invasive region in widely invasive FTCs [29]. Our results corroborate the data shown in these studies, raising the possibility that *POSTN* may play a crucial role in capsular/vascular invasion processes in FTCs.

*DPYSL3*, also known as collapsing response mediator protein 4 (*CRMP4*), is highly expressed in the developing and adult nervous systems [30, 31] and functions in several cellular processes including cell migration, differentiation, and metastasis [32, 33]. Little is known about the role of this gene in thyroid carcinogenesis; however, our results suggest that this gene may play a role in the evolution of an invasive phenotype.

Noteworthy, we reconstructed a branched trajectory by pseudotemporal ordering of how invasive clone cells develop. We observed that clones from the central core of the tumor were annotated as early events, while their spread to the periphery and invasive fronts of the tumor was identified as a late event. This finding strongly supports our hypothesis that the invasive phenotype may be an acquired ability, resulting from a stepwise sequence of events in which various biological processes, including EMT and ECM remodeling interactions, play a crucial role. This analysis has been used recently in thyroid and other types of tumors, showing the temporal dynamics of cancer progression and the invasive pathways, determining key factors contributing to invasion at a high-resolution [34–37].

Moreover, the presence of a *TERT* promoter mutation in our index case allowed us to analyze *TERT* gene expression across the tumor. Interestingly, *TERT*-positive spots were scattered across the tumor tissue, suggestive of a pulsatile expressional pattern of this gene. Similar patterns have been found in *TERT* promoter mutated FTC using *in situ* hybridization, and this puzzling phenomenon requires further investigation [38].

In conclusion, despite the promising findings, this study is not devoid of limitations. The ST analysis is restricted to four different tumor areas of the same patient, making it somewhat hard to draw conclusions for FTCs in general. Moreover, the underlying genetic driver of the index case remains elusive despite extensive NGS and fusion gene analyses. While this study is exploratory and may not have immediate clinical applicability, our findings could prove valuable for subsequent research concerning the invasive potential of thyroid carcinomas. Such insights may hold diagnostic implications for the preoperative assessment of follicular thyroid tumors, wherein markers related to EMT may serve as indicators of invasive potential. Furthermore, recognizing that invasiveness in FTCs is governed by aberrant gene expression patterns, indicating an intensified EMT and ECM interaction, could potentially contribute to identifying molecular networks associated with highly aggressive features and metastatic spread. This, in turn, may open new horizons and facilitate the discovery of anti-tumor drugs tailored for this specific tumor entity.

### Supplementary Information

Below is the link to the electronic supplementary material.

Supplementary file1 Violin plots representing the expression of *DPYSL3* and *POSTN* genes in each cluster. A) Expression of *DPYSL3* in Slide 1 showing high level in clusters 2 and 5; B) Expression of *DPYSL3* in Slide 2 showing high expression level in clusters 6 and 10; C) Expression of *POSTN* in Slide 1 showing the upregulation in cluster 2; D) Expression of *POSTN* in Slide 2 showing high expression level mostly in cluster 10 (TIF 5416 KB)

Supplementary file2 Spatial expression of *TERT*. Expression of *TERT* across the *TERT* promoter mutated tumor. A few spots disseminated across the tumor tissue were highlighted showing an irregular expressional pattern of *TERT *(TIF 21871 KB)

Supplementary file3 (XLSX 10 KB)

Supplementary file4 (XLSX 13 KB)

Supplementary file5 (XLSX 11 KB)

## Data Availability

No datasets were generated or analysed during the current study.
