# Supplementary figures and images for: Spatial Transcriptomics in a Case of Follicular Thyroid Carcinoma Reveals Clone-Specific Dysregulation of Genes Regulating Extracellular Matrix in the Invading Front
Source: Endocr Pathol. 2024 Jan 27;35(2):122–33. doi: 10.1007/s12022-024-09798-0 (PMC11176252; doi:10.1007/s12022-024-09798-0)

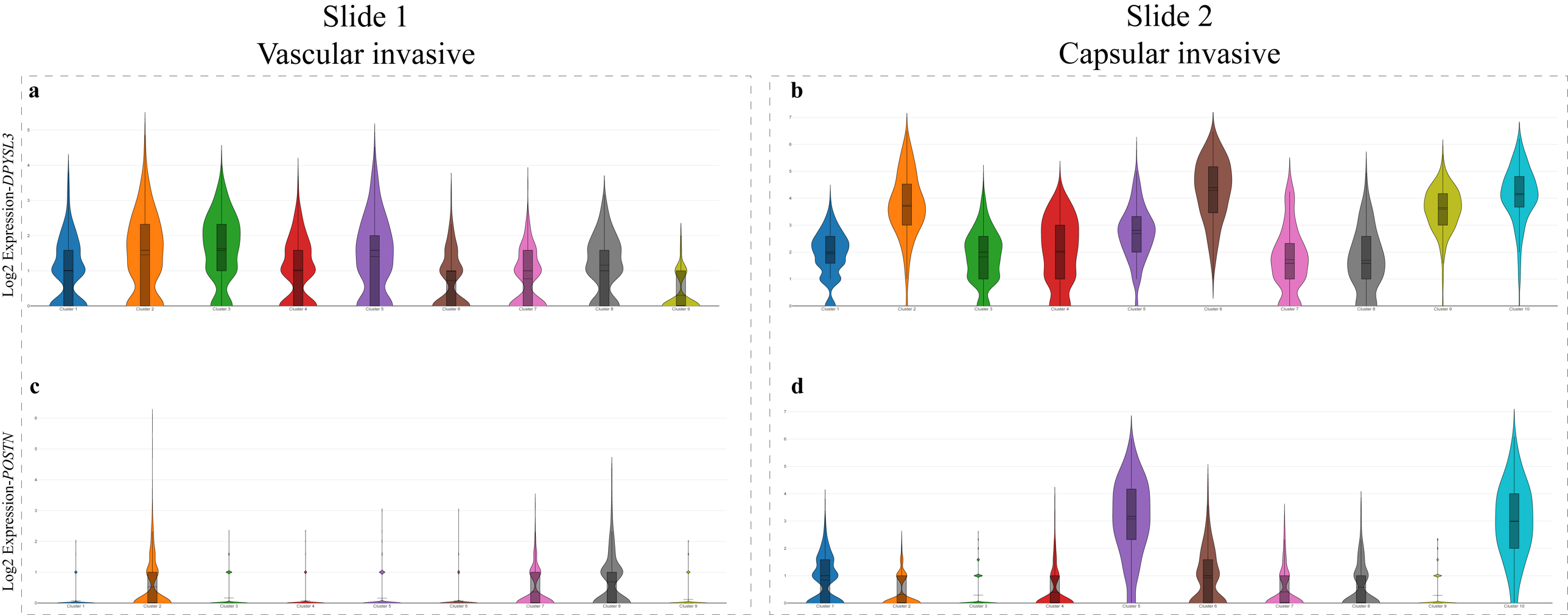

Supplement: Supplementary file 1 — Supplementary file1 Violin plots representing the expression of DPYSL3 and POSTN genes in each cluster. A) Expression of DPYSL3 in Slide 1 showing high level in clusters 2 and 5; B) Expression of DPYSL3 in Slide 2 showing high expression level in clusters 6 and 10; C) Expression of POSTN in Slide 1 showing the upregulation in cluster 2; D) Expression of POSTN in Slide 2 showing high expression level mostly in cluster 10 (TIF 5416 KB) [file 12022_2024_9798_MOESM1_ESM.tif]

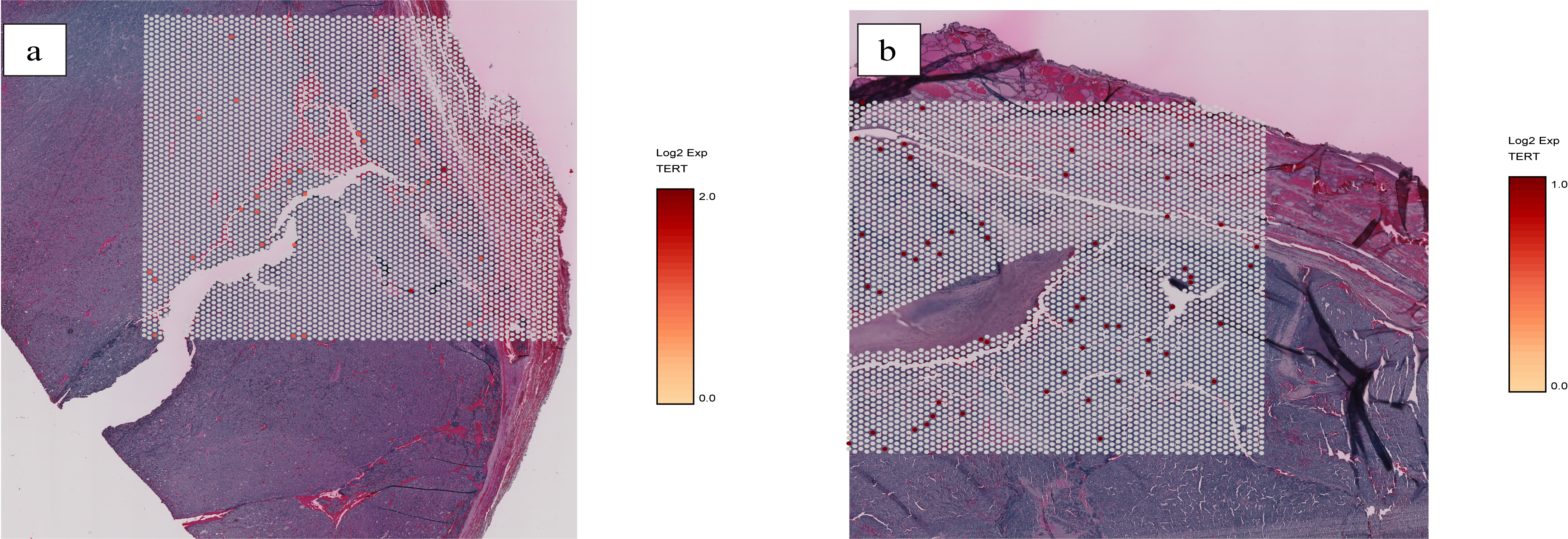

Supplement: Supplementary file 2 — Supplementary file2 Spatial expression of TERT. Expression of TERT across the TERT promoter mutated tumor. A few spots disseminated across the tumor tissue were highlighted showing an irregular expressional pattern of TERT (TIF 21871 KB) [file 12022_2024_9798_MOESM2_ESM.tif]
